# A MULTI-COMPONENT HOME-BASED PHYSICAL THERAPY INTERVENTION FOR IMPROVING COMMUNITY AMBULATION AFTER HIP FRACTURE

**DOI:** 10.1093/geroni/igz038.1565

**Published:** 2019-11-08

**Authors:** Jay S Magaziner

**Affiliations:** University of Maryland Baltimore School of Medicine, Baltimore, Maryland, United States

## Abstract

Presented is a two-group RCT evaluating a multi-component exercise program for hip fracture patients to determine if it is effective in improving the ability to walk independently in the community. Hip fracture patients age ≥60 years (N=210) were assessed and randomized within 26 weeks of hospitalization, and reassessed 16 and 40 weeks later. The primary outcome was ability to walk 300m in six minutes. PUSH (active treatment) included aerobic conditioning, strengthening, balance and functional training. PULSE (attention control) included transcutaneous electrical nerve stimulation, flexibility and active range of motion exercises. Both groups received 2-3 visits per week for 16 weeks in their residences from a physical therapist. 22/96 in PUSH (22.9%) and 18/101 in PULSE (17.8%) (difference 5.1%; 95% CI: -6.1%, 16.3%; P=.37) became community ambulators. We conclude that advancing substantial proportions of hip fracture patients to community ambulation will require more than the intervention evaluated in this study.

